# Safeguarding genomic integrity in beta-cells: implications for beta-cell differentiation, growth, and dysfunction

**DOI:** 10.1042/BST20231519

**Published:** 2024-10-04

**Authors:** Sneha S. Varghese, Alessandro Giovanni Hernandez-De La Peña, Sangeeta Dhawan

**Affiliations:** Department of Translational Research and Cellular Therapeutics, Arthur Riggs Diabetes and Metabolism Research Institute, City of Hope, Duarte, CA 91010, U.S.A.

**Keywords:** beta-cell development, beta-cell maturation, beta-cell senescence, diabetes, DNA damage, genome integrity

## Abstract

The maintenance of optimal glucose levels in the body requires a healthy reserve of the insulin producing pancreatic beta-cells. Depletion of this reserve due to beta-cell dysfunction and death results in development of diabetes. Recent findings highlight unresolved DNA damage as a key contributor to beta-cell defects in diabetes. Beta-cells face various stressors and metabolic challenges throughout life, rendering them susceptible to DNA breaks. The post-mitotic, long-lived phenotype of mature beta-cells further warrants robust maintenance of genomic integrity. Failure to resolve DNA damage during beta-cell development, therefore, can result in an unhealthy reserve of beta-cells and predispose to diabetes. Yet, the molecular mechanisms safeguarding beta-cell genomic integrity remain poorly understood. Here, we focus on the significance of DNA damage in beta-cell homeostasis and postulate how cellular expansion, epigenetic programming, and metabolic shifts during development may impact beta-cell genomic integrity and health. We discuss recent findings demonstrating a physiological role for DNA breaks in modulating transcriptional control in neurons, which share many developmental programs with beta-cells. Finally, we highlight key gaps in our understanding of beta-cell genomic integrity and discuss emerging areas of interest.

## Introduction

The insulin producing pancreatic beta-cells are essential for maintaining systemic glucose homeostasis. Beta-cell failure due to the progressive impairment of cellular identity and function, onset of senescence, and eventual cell-death is central to the pathogenesis of diabetes. Emerging evidence suggests that unresolved DNA damage is a key driver of the spectrum of beta-cell defects observed in diabetes. Failure to repair DNA damage can induce genomic instability and alter genomic functions such as transcription, resulting in a loss of cellular identity, function, and viability. Beta-cells are exposed to changing physiological demands and a variety of stressors throughout their lifespan that can predispose them to DNA damage. This poses a challenge for beta-cells in adult life due to their long lifespan and a limited self-renewal capacity which progressively declines with age. The adult beta-cell reserve is primarily established during early neonatal life via replication and subsequent functional maturation of beta-cells, which are initially differentiate from the pancreatic progenitors (PPs) during embryonic life. The process of replication is inherently vulnerable to DNA damage and involves a stringent monitoring of genomic integrity throughout the cell-cycle. Failure of such mechanisms can impede the expansion, functional maturation, and viability of beta-cells, resulting in a suboptimal beta-cell pool that is unfit to adapt to changing physiological needs. The transition of beta-cells from a replicative, functionally immature state in early life to a post-mitotic, mature state renders them highly vulnerable to the impact of unresolved DNA damage. This scenario warrants beta-cells to implement robust mechanisms to protect their genomic stability to maintain a healthy pool of cells throughout life. However, very little is known about the sources of DNA breaks and the molecular mechanisms that protect beta-cell genomic integrity in maintaining a healthy beta-cell reserve. In this review, we discuss the importance of DNA damage handling in beta-cell health and dysfunction, focusing on the neonatal growth phase as a major window of beta-cell vulnerability. We posit that events during progenitor expansion and differentiation in embryonic life can further influence beta-cell vulnerability. We also highlight current gaps in our knowledge and emerging directions in better understanding the safeguards of beta-cell genomic integrity.

## An overview of genomic integrity: sources of DNA lesions and mechanisms of repair

The optimal functioning of a cell requires stringent preservation of the integrity of genetic material [1]. Cells are exposed to multiple intrinsic and extrinsic agents, such as cellular-stress, aging, and xenobiotics, that can induce DNA lesions. In turn, this can impact genome integrity and genomic output such as replication and transcription and impair cellular function [2–4]. To combat these challenges, cells possess a battery of mechanisms that can sense and repair a variety of DNA lesions, collectively called the DNA damage response (DDR) mechanisms [5–7]. Effective DDR ensures cellular function and viability, healthy progeny upon replication, and protects against mutations and potential transformation. DNA lesions can occur in the form of single or double-strand breaks (SSBs and DSBs, respectively), the former being far more common. SSBs can arise due to spontaneous DNA instability or as intermediates of DNA repair. If not repaired immediately, they can halt transcription and induce deleterious DSBs upon replication [8–10]. The consequences of DNA damage can vary depending on the context, key being the replicative potential of the cell. In replicative cells, DNA lesions can slow or stall the DNA synthesis to cause ‘replication stress’ [11,12]. DNA damage can also lead to mutations or epigenetic changes that can pass on to the daughter cells. This can alter the gene-expression and replication capacity of the daughter cells and even initiate cell transformation [13]. As a safeguard, cell cycle regulation is tightly coupled with DNA damage sensing and repair and involves multiple DNA damage-activated cell cycle checkpoints [13,14]. These checkpoints not only initiate repair but can also induce cell cycle arrest to allow sufficient time for repair. The extent of DNA damage determines whether a cell can effectively repair it and resume normal function or not. If the damage is too severe, cells can assume a state of permanent growth arrest (senescence) or undergo apoptosis to prevent the transmission of the unrepaired damage to daughter cells [15,16]. In addition to these three well recognized sequelae of DNA damage, recent work in multiple models has revealed that DNA breaks can also induce cellular differentiation. Indeed, cell cycle arrest is often a trigger for the differentiation of specialized progenitors during lineage refinement [17,18]. In the terminally differentiated cells that tend to be postmitotic, DNA damage can block transcription and lead to cell dysfunction or death [15]. Effective and timely DDR, therefore, is a pre-requisite for a healthy progenitor pool as well as appropriate differentiation.

**Figure 1.**
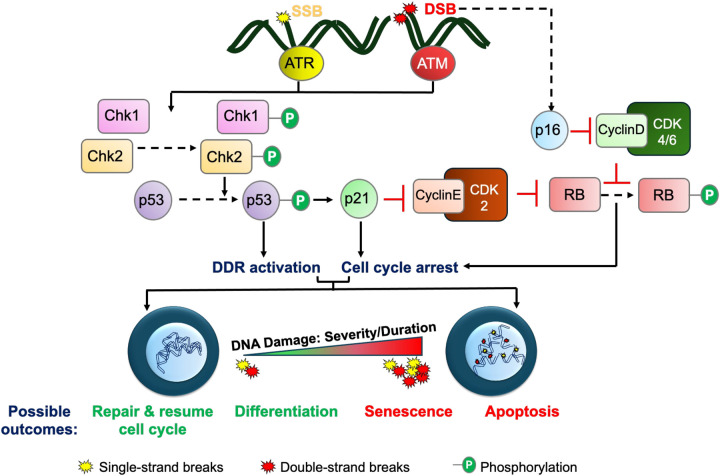
Overview of DNA damage repair and outcomes. DNA breaks induce the activation of ATM (ataxia telangiectasia mutated) and ATR (ataxia telangiectasia and Rad3-related protein) kinases, which results in cell-cycle arrest and activation of DNA Damage Response (DDR) pathways via a signaling cascade involving p53/21 and p16. Depending on the severity and duration of the DNA damage, the cell may eventually repair the damage and resume cell cycle, differentiate, or in more severe cases, become senescent or undergo apoptosis.

DNA damage induces a chain of signaling events designed to pause cell-cycle progression while repairing damage. The ATM (ataxia telangiectasia mutated) and ATR (ataxia telangiectasia and Rad3-related protein) kinases serve as key responders to DNA damage, with ATM responding primarily to DSBs while ATR mainly addresses the SSBs [19] (Figure 1). Activation of ATM and ATR leads to the phosphorylation of the Checkpoint Kinases 1 and 2 (Chk1 and 2), which in turn phosphorylate a variety of cell cycle regulators to inhibit replication. The ATM/ATR and Chk1/2 kinases also phosphorylate p53, which induces the expression of cell cycle inhibitors Cdkn1a/p21, Gadd45, and 14-3-3, further ensuring effective cell cycle arrest. The activation of p53 signaling also induces multiple DNA damage repair pathways to maintain the genomic integrity [16,20]. In contexts such as telomere attrition induced DDR, p16^INK4a^/Rb mediated cell-cycle arrest can supplement the ATR-pathway [21]. The synergy of cell cycle and DNA repair regulation by p53 ensures the maintenance of cell phenotype.

## Persistent DNA damage: a hallmark of beta-cell pathologies

Accumulation of DNA damage is a common early event underlying beta-cell fragility in all forms of diabetes. DNA double stranded breaks and DDR marked by 53BP1 accumulation and p53 activation are observed in beta-cells in rodent models and humans alike in both type 1 and type 2 diabetes (T1D and T2D) [22–24]. Hallmarks of DNA damage and DDR have also been reported in at least one form of monogenic diabetes caused by a missense mutation in the beta-cell transcription factor MafA [25]. Notably, whole-body or abdominal radiation increases the risk of developing diabetes in humans, likely due to radiation related DNA damage in the islets [26]. On the other hand, enhancement of DNA repair mechanisms by agents such as palmitic acid hydroxy stearic acids (PAHSAs) can protect beta-cells in mouse models of T1D [27]. A variety of extrinsic stressors such as inflammation, hyperglycemia, and oxidative stress can induce beta-cell DNA damage [15,28,29]. Circadian misalignment, a significant environmental risk factor for T2D, has also been shown to disrupt the beta-cell DDR [30]. Beta-cells also accumulate DNA damage with age, corresponding to reduced islet function [31,32]. Beta-cells are particularly vulnerable to cellular stress. As professional secretory cells, beta-cells are equipped with high rates of protein synthesis, which increase further in response to increased insulin demand and can induce endoplasmic reticulum (ER)-stress [33,34]. Beta-cells also exhibit high endogenous reactive oxygen species (ROS) production and poor ROS handling capacity, making them susceptible to oxidative-stress [35–37]. Defects in DDR can impair beta-cell stress response and establish a vicious cycle where stress induces DNA damage, which further aggravates stress [38–40]. Beta-cells can also initiate cell-cycle re-entry as a compensatory response to stress [41,42]. While compensatory expansion of adult beta-cells is primarily observed in rodents, attempted beta-cell replication has also been documented in humans with T1D [43]. Such attempts at replication in the face of stress can further aggravate genomic vulnerability and are liable to fail [44,45]. DNA damage triggers the activation of DDR which initially protects against genomic instability. Persistent activation of DDR, however, can lead to onset of cellular senescence, a pro-inflammatory senescence associated secretory phenotype (SASP) and even cell-death [15]. In T1D, the islets proximal to immune cell infiltrate display a more prominent beta-cell DDR, re-affirming the link between chronic DDR and SASP [22]. Overall, the severity and duration of cellular-stress determines whether a beta-cell can successfully repair DNA damage and preserve genomic integrity.

Beta-cell intrinsic factors that influence the DNA repair capacity can also contribute to genomic instability and beta-cell fragility in diabetes. Indeed, genetic polymorphisms in the DNA DSB repair components *XRCC4* and *LIG4* (DNA ligase IV) have been shown to contribute to beta-cell failure in mouse models of T1D [38]. Genetic studies in mice further underscore the significance of DDR in beta-cell health and have revealed context dependent roles of different DNA repair components. For instance, loss of non-homologous end joining (NHEJ) repair component DNA ligase IV in the setting of p53 hypomorphism leads to accumulation of DNA damage, senescence, and reduced beta-cell mass [46]. In contrast, loss of another NHEJ component Ku70 in the same setting cause increased beta-cell replication and islet expansion [47]. These changes are accompanied by the stabilization of islet beta-catenin and increased expression of genes related to cell-cycle progression, suggesting a potential non-NHEJ role for Ku70 in this context [47]. Similarly, mice with beta-cell specific loss of the nucleotide excision repair component *Ercc1* develop adult-onset diabetes [48]. The DNA damage-dependent induction of proinflammatory characteristics in beta-cells relies on ATM signaling and DDR. In agreement, beta-cell specific depletion of ATM protects against islet inflammation induced by Streptozotocin, a beta-cell toxin which is known to induce DNA damage [22]. Nitric oxide (NO), a key mediator of pro-inflammatory cytokine damage to beta-cells in T1D, has dual effects on ATM and ATR signaling [49–51]. Beta-cells up-regulate inducible NO synthase and produce NO in response to cytokines [52]. Prolonged cytokine exposure increases NO levels, inhibiting mitochondrial oxidative metabolism. Unlike most cells, beta-cells lack the metabolic flexibility to increase glycolysis, leading to reduced ATP levels. This ATP reduction indirectly inhibits ATM and ATR signaling, a response unique to beta-cells. Notably, primary islets have lower ATR levels than beta-cell lines, suggesting weaker defenses against SSBs [49].The chromatin modulator YY1 is another key regulator of beta-cell DDR and is depleted in beta-cells from individuals with T2D [39]. Mutations in *YY1* have also been linked with insulinomas [53]. The loss of beta-cell genomic stability is also observed in congenital hyperinsulinism (CHI) resulting from mutations that cause glucokinase (GCK) activation. GCK activation induces beta-cell replication, and eventually results in DSBs, p53 activation, and cell-death [23]. Increased glycolysis due to GCK activation results in increased ATP production and chronic membrane depolarization, which is necessary and sufficient to induce the DNA damage observed in GCK-CHI [23]. Collectively, these studies show that failure to repair DNA damage can be detrimental for beta-cell health.

## Beta-cell development: a phase of high genomic vulnerability

**Figure 2.**
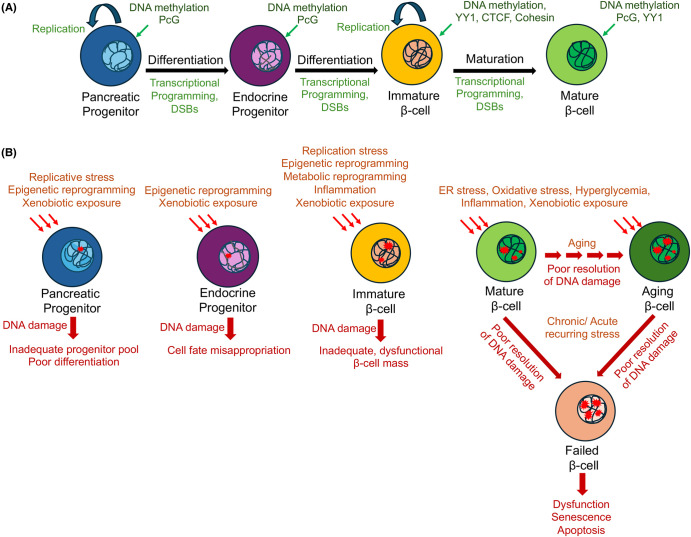
Inducers and consequences of DNA damage in beta-cell homeostasis. (a) Sources of DNA double strand breaks (DSBs) across the life span of a beta-cell. The processes of replication, transcriptional programming, and the associated epigenetic changes during beta-cell differentiation, growth, and maturation can introduced DNA breaks. Beta-cells are also exposed to a variety of stressors throughout their lifespan that also can induce DNA breaks. Programmed DSBs can assist transcriptional changes and promote differentiation. (b) Defects in repair of physiological DNA breaks and/or stress (chronic stress or multiple exposures to acute stress) can cause an increased incidence and accumulation of DNA damage, which if unresolved, can have stage-specific deleterious consequences for beta-cell differentiation and growth, compromise beta-cell health, and increase the risk for beta-cell failure.

Establishment of the beta-cell reserve is a multi-step process which begins in the embryonic life with progressive cell-fate refinement within the endodermal lineage to generate beta-cells [54]. The PPs are self-renewing cells that need to exit cell-cycle to differentiate into the committed endocrine progenitors (EPs) [55] (Figure 2a). The EPs then give rise to the different endocrine cell types in the embryonic pancreas, including beta-cells [54,56–59]. This is followed by a wave of beta-cell expansion by replication in the neonatal growth phase (until weaning in mice, <5 years in human) [41,60–63]. Neonatal beta-cells are, however, functionally immature and lack responsiveness to fluctuations in glucose levels [64–67] (Figure 2a). As the growth phase progresses, beta-cells gradually exit the cell-cycle and transition to functional maturity and acquisition of glucose responsiveness, which involves metabolic reprogramming triggered by the establishment of circadian feeding/fasting cycles [68–73]. Thus, beta-cell differentiation involves the precise coordination of cellular self-renewal *vs.* differentiation throughout differentiation, including the PP and neonatal beta-cell stages. This requires a cascade of stepwise instructive epigenetic changes that guide the developing cells toward progressive specialization. DNA methylation is a key epigenetic module involved in multiple steps of beta-cell differentiation [74]. For instance, maintenance of existing DNA methylation patterns is essential for the survival of PPs as they exit cell-cycle and differentiate into the endocrine lineage [75]. Additionally, PPs utilize heterogeneous DNA methylation patterns to establish selective competence for distinct endocrine cell fates [76]. Preserving these patterns in the differentiated endocrine cells is essential for maintaining their unique cellular identity [77]. DNA methylation patterning also directs the transition of beta-cells from a replicative to a functionally mature state in neonatal life [69]. Patterning of the cell-type specific chromatin context and architecture is also crucial for the differentiation and maturation of beta-cells. Indeed, modulators of the 3D chromatin architecture, such as the CCCTC binding factor (CTCF), Cohesin complex, and Yin-Yang 1 (YY1), as well as the Polycomb group (PcG) proteins play an important role in protecting genomic integrity and beta-cell homeostasis [39,78–87].

The cycles of replication and epigenetic programming that occur throughout beta-cell differentiation and maturation, can create multiple windows of genomic vulnerability (Figure 2b). First, the process of replication can generate DNA breaks, which if unrepaired, can impede cell expansion and differentiation [11,12]. The initiation and efficacy of DDR depends upon the chromatin context; the modulation of chromatin organization safeguards genomic stability to ensure successful replication and transcription during different phases of the cell-cycle [88,89]. Defects in modulation of chromatin structure can therefore impede DNA repair and compromise genome integrity. For example, loss of *Yy1* in the developing beta-cells leads to impaired beta-cell functional maturation and loss of beta-cell viability [39]. Emerging work suggests that the temporal regulation of Cohesin complex recruitment not only mediates the transcriptional control underlying beta-cell maturation but is also essential for maintaining the genomic integrity of developing beta-cells [79,81]. Second, the inherent plasticity of epigenetic states, e.g. the reversible nature of DNA methylation, not only facilitates differentiation but can also introduce genomic vulnerability. DNA demethylation involves progressive oxidation of 5-methylcytosine (5mC) at CpG dinucleotides results in the formation of 5-hydroxymethylcytosine (5hmC), 5-formylcytosine (5fC), and 5-carboxylcytosine (5caC). 5fC and 5caC are then glycosylated by thymine DNA glycosylase and targeted for BER, resulting in the conversion of 5mC to cytosine [90,91]. Modulation of DNA methylation patterns during differentiation, therefore, can introduce DNA lesions. Failure to maintain DNA methylation patterns during DNA replication can also induce genomic instability [92,93]. Recent data show that PPs have very low genomic 5hmC content, which progressively increases with cell-cycle exit during progenitor differentiation and beta-cell functional maturation [94]. However, it remains to be seen whether the 5hmC enrichment observed during beta-cell differentiation and maturation is associated with DNA breaks and repair. Recent data show that epigenetic patterning during the PP stage also directs the transcriptional programming of the postnatal beta-cell heterogeneity and function [95]. Any unresolved DNA breaks that occur in early beta-cell development can therefore have a profound effect on the mature beta-cell phenotype.

In addition to replication and epigenetic reprogramming, the extensive metabolic changes associated with the process of beta-cell maturation may further exacerbate DNA damage vulnerability during the growth phase (Figure 2b). For instance, the changes in oxidative metabolism and redox homeostasis that occur during beta-cell maturation may alter ROS levels [96]. This can, in turn, potentially trigger oxidative DNA damage by introducing ROS-mediated DNA lesions, 8-oxoguanine (8-oxoG) and 8-oxo-2′-deoxyguanosine (8-oxodG) [97]. Failure to repair DNA lesions during the growth phase may not only impede beta-cell expansion but can also compromise their functional maturation and viability of beta-cells. This can result in an inadequate reserve that is not well-equipped to respond to changing physiological demands and multiple stressors that beta-cells encounter throughout life (Figure 2b).

## Physiological DNA breaks: lessons from neurons

Despite originating from different germ layers, beta-cells share many characteristics with neurons, including shared developmental transcriptional regulation and use of membrane depolarization for secretion in response to stimuli. For instance, the transcription factors Neurogenin 3 (Neurog3) and NeuroD1 are both essential for brain and islet development [98,99]. Similarly, the RE-1 Silencing Transcription factor is expressed in most cells except mature neurons and beta-cells [100,101]. Moreover, islets harbor beta-cells that express select neuromodulatory molecules and peptides such as Tyrosine Hydroxylase and Neuropeptide Y in a context dependent manner [95,102,103]. Neurons display a very high vulnerability to DNA breaks. Recent work on stimulus-induced DNA breaks and their role in transcriptional control in neurons could therefore inform our understanding of the sources of DNA breaks in beta-cells.

DNA damage has long been viewed as a stochastic ‘wear and tear’ process that occurs in response to multiple stressors and is typically considered harmful for cell viability [8]. However, emerging evidence shows that programmed DNA breaks in the regulatory regions of the genome, coupled with the activation of DDR, may be instrumental in signal-dependent transcriptional regulation [104]. These data argue that DNA repair not only repairs the stochastic damage but is also an important mechanism that regulates genomic architecture and function [105]. Programmed DNA breaks can be introduced upon the topoisomerase-dependent resolution of topological stress during transcription and Cohesin-mediated loop extrusion [106–109]. SSBs and DSBs are a part of the topoisomerase cleavage complex that are re-joined upon completion of the reaction. However, failure to complete this process can result in persistent breaks that can invoke a DDR [110]. BER is another mechanism that can introduce programmed SSBs or DSB towards highly targeted genomic modification. This is exemplified by lymphocytes where base deamination is used to generate uracil, which introduces SSBs via BER. This strategy allows the immune system to adapt to new challenges by supporting antibody class switching via somatic hypermutation [111]. As discussed earlier, programmed BER also contributes to epigenetic modulation via DNA demethylation, which plays a critical role in embryogenesis and cell fate specification [90,91].

Neurons display a very high frequency of programmed DNA breaks which occur at specific sites in the genome. Genome-wide profiling shows neuronal enhancers display high levels of SSBs at or near CpG dinucleotides. These SSBs and DNA repair synthesis sites are enriched for 5hmC and 5fC. The positioning of these breaks indicates their non-stochastic nature and relevance to epigenetic programming. Relevant to this, postmitotic neurons have the highest levels of 5hmC compared with any other cell type [112]. From a phenotypic perspective, neuronal activation has been well documented to generate DSBs at specific loci [113–115]. These regions occur in the promoters of early response genes (ERGs) that mediate adaptive changes in synapses and memory [116]. The expression patterns of ERGs upon neuronal stimulation align well with the formation and repair of activity induced DSBs, suggesting a physiological role of this process in transcriptional adaptation [104]. On the flip side, DNA breaks induced via topoisomerase and BER mechanisms have also been shown to modulate neural transmission and plasticity in the context of glutamatergic neurons [107,116]. Mounting evidence suggests the involvement of programmed DSBs and their repair in long-term memory processes. Interestingly, this involves the recombination-activating genes that mediate antibody class switching in lymphocytes [117]. A recent study shows that learning induced DSBs and subsequent TLR9-mediated repair are involved in the recruitment of specific hippocampal neuronal clusters to memory circuits [118]. Given this background, it is not surprising that the neuronal genome is especially vulnerable to impairments in DNA repair. Indeed, neurons have a high propensity for abortive topoisomerase reactions and defects in repairing the topoisomerase- induced SSBs and DSBs have deleterious consequences for neurons, contributing to a variety of genetic neuronal disorders and neurodevelopmental delays [107,119]. Unrepaired DNA breaks have also been linked with multiple neurodegenerative disorders [120]. Factors such as their long lifespan with minimal regenerative capacity, elevated transcriptional demand, and a bias for long transcripts, high oxidative metabolism, and poor handling of ROS may additionally contribute to neuronal susceptibility to DNA breaks [107]. Beta-cells share many of these characteristics with neurons, and this similarity is further underscored by their high genomic 5hmC content [94,98,99]. While beta-cells are also highly vulnerable to DNA breaks, it remains to be seen whether they leverage programmed DNA breaks for modulating transcriptional responses during physiological processes such as beta-cell differentiation, maturation, and glucose responsive insulin secretion.

## Knowledge gaps and opportunities

We identify three key factors that could contribute to beta-cell vulnerability to DNA breaks: (1) developmental processes, (2) beta-cell intrinsic and extrinsic factors, and (3) programmed DNA breaks. Addressing these aspects could clarify the causal role of DNA damage in driving beta-cell failure in diabetes.

1. *Developmental processes*: Pancreatic beta-cells, with their high secretory burden and oxidative metabolism, are highly susceptible to cellular stress and DNA breaks. Replication and the epigenetic and metabolic reprogramming during beta-cell differentiation can also introduce DNA breaks, their impact exacerbated by the shift to a quiescent, long-lived phenotype upon maturation. DNA breaks also progressively accumulate with aging [3,4]. These lingering DNA lesions have the potential to alter the cellular identity, function, and survival of mature beta-cells, and can impair their adaptation to changing metabolic needs. The extent of DNA damage may also shape cell-fate choices during beta-cell differentiation [15]. Cells with modest DNA damage may temporarily pause the cell-cycle to repair the damage and either resume cell cycle or differentiate, depending on the context. In contrast, cells with extensive DNA damage could launch a pro-inflammatory senescence response to facilitate their clearance by the immune cells. Unresolved DNA damage during development may thus impact beta-cell immune surveillance and immunogenicity. This is a tantalizing hypothesis, given that the peak incidence of beta-cell autoimmunity occurs in early life and during puberty, periods of significant beta-cell stress and turnover. It is plausible that unresolved DNA damage in the setting of genetic predisposition to T1D can exacerbate disease risk. Similarly, impaired DNA repair can predispose to beta-cell defects and contribute to T2D.
2. *Beta-cell intrinsic and extrinsic mechanisms*: The heterogeneous nature of beta-cells, particularly in their replicative capacity [121], suggests that different beta-cell subtypes may have varying DNA repair capacities. Other intrinsic factors, such as polymorphisms in DNA repair pathways and beta-cell-specific enhancers, may further influence the repair capacity and epigenetic reprogramming potential of beta-cells and determine their response to stress. This variability can explain the heterogeneous vulnerability of beta-cells in diabetes, where an imbalance between DNA damage load and repair capacity might drive beta-cell failure. Such imbalance could arise from either inadequate repair in response to physiological levels of stress or chronic/excessive stress that exceeds normal repair capacity. Extrinsic factors, like impaired immune-mediated clearance of senescent cells, can also exacerbate beta-cell stress, increasing repair demands. Other specialized, long-lived cell types are also susceptible to unresolved DNA damage. Thus, unresolved DNA damage in non-beta islet cells and islet-resident immune, vascular, and neuronal cells can directly contribute to islet pathology and indirectly impact beta-cell function. Emerging evidence suggests that alpha-cells in T1D, though dysfunctional, do not undergo senescence unlike beta-cells [122,123]. Differences in overall repair capacity and turnover likely determine the distinct stress responses among islet cell types.
3. *Programmed DNA breaks*: Programmed DNA breaks may activate specific developmental enhancers to refine beta-cell subtype choices and also modulate the inducible transcriptional programs involved in beta-cell response to various physiological stimuli and/or stress. Environmental factors such as nutrient quality, feeding-fasting behavior, and circadian entrainment that guide the beta-cell response likely also influence beta-cell genomic integrity [30,124]. Increased demand for inducible transcriptional programming due to environmental changes and poor resolution of consequent programmed breaks could then result in DNA damage and impair beta-cell homeostasis.

Recent advances in single-cell and spatial transcriptomics, chromatin state, and proteomic profiling, alongside temporal assessment of DNA damage across cell-types in response to stress or at different stages of diabetes development, could help address these questions. How the DNA break programming intersects with chromatin topology to modulate beta-cell transcriptional response to physiological changes also warrants a comprehensive investigation. Understanding the molecular mechanisms of DNA break management can clarify the triggers of beta-cell failure and explain the developmental basis of diabetes risk. Such work would also inform therapeutic approaches for producing and expanding mature human beta-cells.

## Perspectives

- Beta-cells are highly susceptible to DNA damage, given their high metabolic activity and secretory burden to meet the body's insulin demands, exacerbated by their long-lived and largely post-mitotic nature.
- Compromised genomic integrity due to unresolved DNA damage is a key early driver of beta-cell defects in diabetes. Emerging evidence reveals beta-cell growth as a phase of heightened vulnerability to DNA damage.
- Future work identifying the sources and consequences of DNA damage in beta-cells, such as programmed DNA breaks during transcriptional adaptation in response to changing metabolism, will be crucial for understanding the roots of beta-cell failure.

## Abbreviations

ATM: ataxia telangiectasia mutated
CHI: congenital hyperinsulinism
DDR: DNA damage response
DSB: double-strand break
EP: endocrine progenitor
ERG: early response genes
GCK: glucokinase
NHEJ: non-homologous end joining
NO: nitric oxide
PP: pancreatic progenitor
ROS: reactive oxygen species
SASP: senescence associated secretory phenotype
